# SARS-CoV-2 Vaccination Response in Japanese Patients with Autoimmune Hepatitis: Results of Propensity Score-Matched Case–Control Study

**DOI:** 10.3390/jcm12165411

**Published:** 2023-08-20

**Authors:** Kei Moriya, Tomoko Nakakita, Natsuki Nakayama, Yuya Matsuo, Yusuke Komeda, Junichi Hanatani, Daisuke Kaya, Shinsaku Nagamatsu, Hideki Matsuo, Masakazu Uejima, Fumihiko Nakamura

**Affiliations:** 1 Department of Gastroenterology, Nara Prefecture General Medical Center, Nara 630-8581, Japan; jean.mat@outlook.jp (Y.M.);; 2 Department of Gastroenterology, Nara Medical University, Nara 634-8521, Japan; 3 Department of Laboratory Medicine, Nara Prefecture General Medical Center, Nara 630-8581, Japan; 4 Department of Diabetes and Endocrinology, Nara Prefecture General Medical Center, Nara 630-8581, Japan

**Keywords:** autoimmune hepatitis, case–control studies, COVID-19, propensity score, SARS-CoV-2, vaccination

## Abstract

Background/Aims: Although the World Health Organization declared the end of the public health emergency of international concern focusing on COVID-19 in May 2023, this bothersome virus continues to mutate, and the possibility of the emergence of mutant strains with high infectivity and severe disease rates has not disappeared. Thus, medical evidence must be accumulated, which is indispensable for protecting both patients under immunosuppressive treatments and the healthy population. This study examined SARS-CoV-2 vaccination responses in Japanese patients with autoimmune hepatitis (AIH) compared with healthy controls. Methods: This observational study registered 22 patients with histologically diagnosed AIH and 809 healthy controls in our hospital. Their Elecsys anti-SARS-CoV-2 spike antibody concentrations before and after vaccination were evaluated. Results: In this study, 72.7% and 18.2% of patients with AIH received steroids and azathioprine, respectively. Significant negative correlations were found between age and anti-SARS-CoV-2 spike antibody concentration in both groups; however, no sex differences were found. Although anti-SARS-CoV-2 spike antibody concentration was drastically augmented after the second vaccination (*p* < 0.05) in the AIH group, these levels were significantly lower than those in the controls (*p* < 0.05). In the age- and sex-matched analysis, the population ratio with a minimum response (≤100 binding antibody units (BAU/mL) was higher among patients with AIH than among controls 26 weeks after the second vaccination (44% vs. 7%, *p* < 0.05). Conclusions: The anti-SARS-CoV-2 spike antibody concentration in AIH patients was significantly lower than that in controls after the second vaccination. Continued and widespread vaccination, particularly for patients requiring medical immunomodulation, is recommended.

## 1. Introduction

The incidence of autoimmune hepatitis (AIH), a chronic inflammatory liver disease with unknown etiology, is increasing worldwide [1,2], and with the arrival of a full-fledged aging society, the proportion of elderly patients with autoimmune hepatitis is steadily increasing in Japan [3]. In patients with AIH, life prognosis is threatened by the potential persistence of the disease and development of liver fibrosis [4]. Thus, from a physical point of view, good control of liver fibrosis progression is very important in the pathological management of AIH [5]. In addition, Takahashi et al. reported that patients with AIH showed impaired health-related quality of life, which was associated with not only disease progression but also comorbid diseases and treatment [1]. Thus, given that immunosuppressive therapy is necessary to control the pathogenesis of AIH, the risk of infectious disease exacerbation, including the development of opportunistic infections, is undoubtedly present in patients with AIH.

According to the World Health Organization (WHO), as of June 2023, more than 750 million infections and approximately 7 million deaths have been reported worldwide with respect to COVID-19 [6]. On the contrary, the WHO declared the end of the public health emergency of international concern in early May 2023 after the majority of the population in many countries and regions, including Japan, had been vaccinated a prescribed number of times and mass immunity had increased, resulting in a decrease in the fatality rate of COVID-19 [7]. However, this does not mean the “end of this bothersome virus.” Since COVID-19 continues to mutate and the possibility of the emergence of mutant strains with high infectivity and rates of severe disease has not disappeared, the importance of a surveillance system that can respond quickly and appropriately to such an eventuality will not change. WHO, in its press conference at the time of declaring the emergency over, also stated that the actual number of deaths due to COVID-19 was at least 20 million [7], and that continued vaccination, particularly for those with the disease, is strongly recommended in the future. In fact, the mortality rate from COVID-19 infection in elderly patients is significantly higher than in non-elderly populations, and patients who are continuously taking immunosuppressive drugs such as steroids have a higher rate of severe disease when infected with COVID-19 [8]. Compounding these aforementioned facts, patients with autoimmune hepatitis in Japan seem to be particularly threatened by the risk of severe disease due to COVID-19 infection.

The first mRNA vaccine in human history, which was rapidly developed in response to the COVID-19 pandemic, is attracting attention as a useful tool to avoid severe COVID-19 infection [9,10]. However, the incidence of adverse reactions at the time of vaccination with this vaccine is higher than with conventional vaccines, and naturally, there is a lack of firm evidence regarding its long-term safety. Therefore, there is no small need for clinicians to establish useful clinical indicators to guide their decisions in encouraging patients for initial vaccination or revaccination. It is true that research has been limited on vaccine antibody titers for autoimmune diseases, a condition for which long-term steroid use and other immunosuppressive therapies constitute the basic treatment. AIH is the second most common autoimmune liver disease after primary biliary cholangitis [11], and immunosuppressive therapy is essential for its pathogenetic control [5]. 

In this study, we examined SARS-CoV-2 vaccination responses in Japanese patients with AIH and compared them with the results of healthy medical workers using propensity score-matched analyses. 

## 2. Research Design and Methods

### 2.1. Study Population and Data Collection

In this study, 22 Japanese patients with histologically diagnosed AIH who attended our hospital between May 2021 and March 2022 based on the international AIH criteria suggested by the International Autoimmune Hepatitis Group [12] were voluntarily enrolled. They were generally treated with adrenal steroids and/or immunomodulator and were in their clinical remissions, defined as having serum alanine aminotransferase levels below the normal upper limit for at least 6 months. In these 22 patients with AIH, five patients had diabetes mellitus and three with hypothyroidism. Patients with hepatitis B virus, hepatitis C virus, human immunodeficiency virus, and a history of excessive alcohol consumption were excluded. Those with some malignancies were also excluded. A total of 840 healthy medical employees working in our hospital were initially registered as healthy controls, and 31 of them were then excluded for testing positive for serum SARS-CoV-2 nucleocapsid protein, which indicated a previous history of COVID-19. In accordance with the product instruction manual of Elecsys^®^ Anti-SARS-CoV-2 (Roche Diagnostics Cor., Indianapolis, IN, USA), cases with antibody titers of 1.0 or higher were considered positive. Finally, 809 healthy controls were enrolled in this study (control group).

All study participants were vaccinated with BNT162b2 mRNA COVID-19 vaccine (Pfizer-BioNTech, New York, NY, USA)©. General information about healthy controls, patients, and types and doses of immunosuppressants were collected. In the control group, blood sampling was performed before the first vaccination and 12/26 weeks after the second vaccination. In the AIH group, blood sampling was performed before the first vaccination, 3 weeks after the first vaccination, and 4/12/26 weeks after the second vaccination.

### 2.2. Laboratory Assessments

Two different types of antibodies for SARS-CoV-2 were used in this study. One was Elecsys^®^ Anti-SARS-CoV-2 S and the other was Elecsys^®^ Anti-SARS-CoV-2 (Roche Diagnostics Cor., IN, USA). The former is for the quantitative determination of antibodies to the SARS-CoV-2 spike protein and is useful not for assessing past SARS-CoV-2 infection but for assessing the effect of SARS-CoV-2 vaccination. The latter is for the quantitative determination of antibodies to the SARS-CoV-2 nucleocapsid protein and is useful not for assessing the effect of SARS-CoV-2 vaccination but for assessing the past SARS-CoV-2 infection. Serum concentrations of these antibodies were measured in our laboratory using the electrochemiluminescence immunoassay method.

### 2.3. Statistical Analyses

Numerical variables were expressed as medians and quartiles. The Mann–Whitney U test was adopted to examine the significant difference between two groups without a normal distribution. Unrelated categorical variables were evaluated by using Pearson’s chi-square test. Correlation was assessed using Spearman’s rank correlation coefficients. Values of *p* < 0.05 were considered statistically significant. JMP version 14.3 (SAS Institute Inc., Cary, NC, USA) was used for statistical analyses.

### 2.4. Ethical Issues

The Ethical Committee of Nara Prefecture General Medical Center and Nara Medical University approved this study (Approval nos. 421 and 739), which was conducted according to ethical principles and Japanese ethics guideline for life science and medical research involving human subjects [https://www.mhlw.go.jp/content/000769923.pdf. accessed on 30 June 2023]. This study was conducted according to the Declaration of Helsinki, and informed consent was obtained via an opt-out method. 

## 3. Results

### 3.1. Clinical Characteristics of the Study Population

The clinical profiles of the 809 controls and 22 patients with AIH enrolled in this study are shown in Table 1. The median age of the AIH group was 65.0 years, which made it significantly older than the control group (*p* < 0.01). In the AIH patient group, some patients who had been diagnosed as AIH in the past and continuously treated for two or three decades were also included in the study. The male-to-female ratio of the AIH group was 4/18, which was not significantly different from that (186/623) of the control group (*p* = 0.79). In the AIH group, 72.7% (16/22) of the patients were treated with adrenal steroids, and the median daily dose of prednisolone was 5.5 mg. Azathioprine was prescribed in 18.2% (4/22), with a median dose of 75.0 mg per day. None of the patients with AIH received anti-allergic agents. The clinical data of controls were not supplied because not all the participants in the control group had their medical records in our hospital and detailed information on steroids, azathioprine, and anti-allergic drugs were not able to be collected.

### 3.2. Risk Factors for Attenuated Humoral Vaccination Response in the Study Population

**Figure 1:**
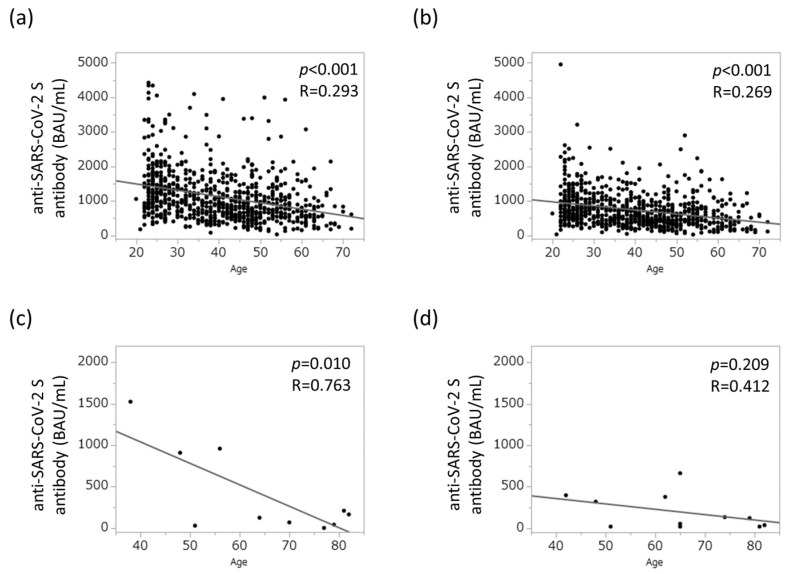
Relationship between aging and humoral vaccination response in the study population. (**a**) Humoral vaccination response in healthy controls 12 weeks after the second vaccination. (**b**) Humoral vaccination response in healthy controls 26 weeks after the second vaccination. (**c**) Humoral vaccination response in patients with AIH 12 weeks after the second vaccination. (**d**) Humoral vaccination response in patients with AIH 26 weeks after the second vaccination. AIH, autoimmune hepatitis; BAU, binding antibody units.

As shown in Figure 1a,b, a clear negative correlation was found between age at vaccination and anti-SARS-CoV-2 spike antibody concentration in the control group (*p* < 0.001 and *p* < 0.001, respectively). A similar negative correlation was observed in the AIH group (*p* < 0.001 and *p* = 0.209) (Figure 1c,d, respectively). Conversely, no sex differences were found with respect to anti-SARS-CoV-2 spike antibody concentration (Supplementary Figure S1a,b).

### 3.3. Attenuated Humoral Immune Response in the AIH Group

**Figure 2:**
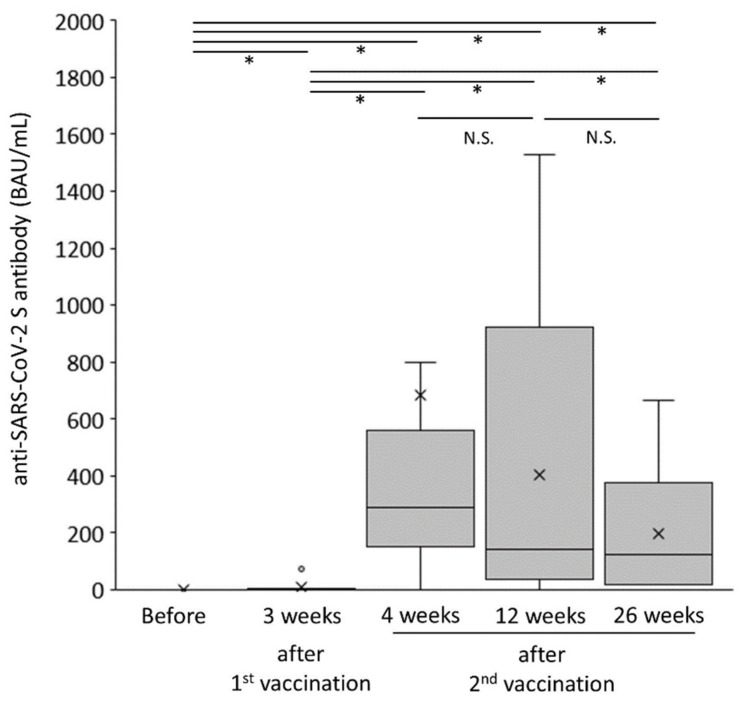
Changes in humoral vaccination response over time in patients with autoimmune hepatitis. * *p* < 0.05. BAU, binding antibody units; NS, not significant.

**Figure 3:**
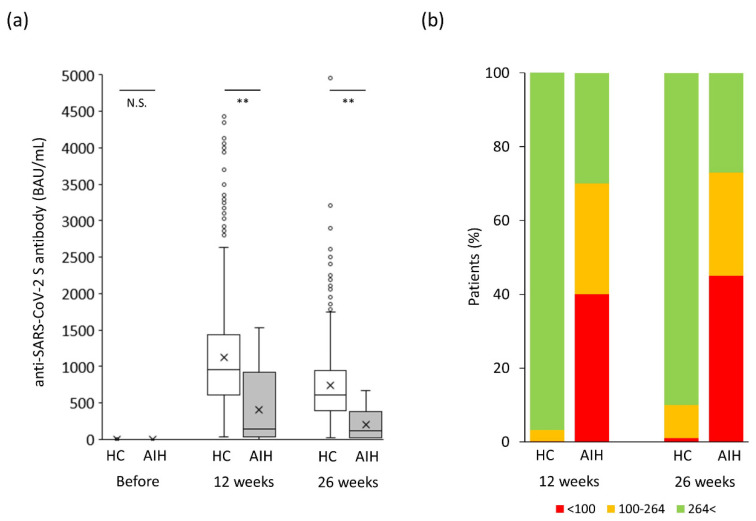
Changes in humoral vaccination response over time in the *original* study population. (**a**) Humoral vaccination response over time in the study population. (**b**) Distribution of antibody levels in the study groups based on the cutoff for “minimum response” (<100 BAU/mL) and “tolerable response” (≤264 BAU/mL). AIH, autoimmune hepatitis; BAU, binding antibody units; HC, healthy controls; NS, not significant. ** *p* < 0.005.

The changes in humoral vaccination response over time in the AIH group are shown in Figure 2. The anti-SARS-CoV-2 spike antibody concentration was drastically augmented just after the second vaccination (*p* < 0.05) and tended to gradually decrease (*p* = 0.08). As shown in Figure 3a, the anti-SARS-CoV-2 spike antibody concentration in the AIH group was significantly lower than that in the control group at both 12 and 26 weeks after the second vaccination (*p* < 0.005 and *p* < 0.005, respectively). 

Given that the predictive degree of immunity to SARS-CoV-2 based on antibody levels has not been determined yet [13], the cutoff for a “minimum response” was set at 100 BAU/mL in the present study based on previous reports [14,15,16,17]. An additional cutoff for a “tolerable response” was set at 264 BAU/mL based on a previous report [18]. Both endpoints, “minimum” and “tolerable” responses, significantly occurred more frequently in the AIH group than in the control group 12 weeks after the second vaccination (minimum response: 40% vs. 0.2%, *p* < 0.0001; tolerable response: 70% vs. 3.5%, *p* < 0.0001; Figure 3b). Twenty-six weeks after the second vaccination, both endpoints, “minimum” and “tolerable” responses, also occurred more frequently in the AIH group than in the control group (minimum response: 45% vs. 1%, *p* < 0.0001; tolerable response: 73% vs. 10%, *p* < 0.0001; Figure 3b). 

### 3.4. Propensity Score-Matched Analysis

**Figure 4:**
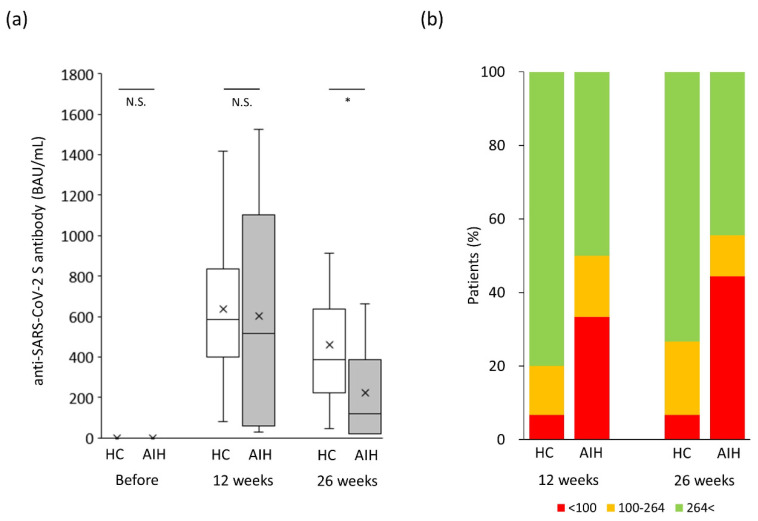
Changes in humoral vaccination response over time in the *propensity-matched* study population. (**a**) Humoral vaccination response over time in the study population. (**b**) Distribution of antibody levels in the study groups based on the cutoff for “minimum response” (<100 BAU/mL) and “tole response” (≤264 BAU/mL). AIH, autoimmune hepatitis; BAU, binding antibody units; HC, healthy controls; NS, not significant. * *p* < 0.05.

To exclude the effect of age, which was a major confounding factor in this study, a propensity score-matched analysis was performed. As a result, 15 patients were finally selected from each of the AIH and control groups (Table 1), and their median ages were 61.0 and 60.0 years, respectively (*p* = 0.86). The male-to-female ratio in the control group was 2/13 and was not significantly different from the value of 3/12 in the AIH group (*p* = 1.00). In the AIH group, 73.3% (11/15) of the patients were treated with prednisolone, and its median daily dose was 6.0 mg. Immunomodulator was prescribed to 20.0% (3/15) of the patients, and its median dose was 75.0 mg. As shown in Figure 4a, the anti-SARS-CoV-2 spike antibody concentration in the AIH group was significantly lower than that in the control group 26 weeks after the second vaccination (*p* < 0.05). Both endpoints, “poor” and “tolerable” responses, in the AIH patient group occurred more frequently than in the control group 12 weeks after the second vaccination (minimum response: 33% vs. 7%, *p* = 0.48; tolerable response: 50% vs. 20%, *p* = 0.17; Figure 4b), despite the lack of significant difference due to the insufficient number of cases analyzed. Moreover, 26 weeks after the second vaccination, the “minimum” response was reported significantly more frequently in the AIH group than in the control group (minimum response: 44% vs. 7%, *p* = 0.03; Figure 4b), although the tolerable response in the AIH group occurred about twice as often as in the control group (tolerable response: 56% vs. 27%, *p* = 0.16; Figure 4b) without a significant difference.

## 4. Discussion

One of the therapeutic measures rapidly created by the collective wisdom of mankind against this virulent virus, COVID-19, involved the two kinds of mRNA vaccines [9,10]. These vaccines safely induced anti-SARS-CoV-2 immune responses in healthy participants and reduced the mortality rates in patients with cirrhosis [19].

In Japan, BNT162b2 mRNA COVID-19 vaccine (Pfizer-BioNTech) © was approved in February 2021, and in this study, the anti-SARS-CoV-2 spike antibody concentration after vaccination was markedly lower in the AIH group than in the control group. In addition, after 3 months of repeated vaccinations, the majority of patients already had antibody titers at levels considered clinically inadequate. Patients with AIH are generally ubiquitous among middle-aged and older women, and the continuation of immunomodulating agents such as adrenal steroids and thiopurines is essential for disease control [20,21]. In older patients and patients with immunosuppressants, relatively low anti-SARS-CoV-2 spike antibody concentrations have already been reported in other diseases [8,22], and the results of this study were also consistent with these reports.

Several cases of AIH-like liver injury have been noted after COVID-19 vaccination [23,24,25,26], and its pathogenesis was reported to be caused by the hyperstimulation of the immune system and molecular mimicry with human self-components [27]. However, a recent report based on the results of various sequencing analyses, including gene expression profiling, suggests that these are not true instances of AIH but belong to a new category of vaccine-induced liver injury [28], and another report showed real-world data in that COVID-19 vaccine did not increase AIH risk [29]. Efe et al. reported that treatment with systemic glucocorticoids or thiopurines before COVID-19 onset in patients with AIH was significantly associated with COVID-19 severity [30] and that SARS-CoV-2 vaccination significantly reduced the risk of COVID-19 severity and mortality in patients with AIH [31]. Compared with other autoimmune liver diseases such as PBC and PSC, the immune response after vaccination is weaker in AIH [13]. The number of reports on the variation of antibody titers over time following the COVID-19 vaccination of patients with AIH remains small and the number of eligible patients is limited [32,33]. Our findings will also be recognized as a new achievement because no studies have examined the percentage of patients with AIH who maintain optimal antibody titers after vaccination and can clinically benefit from vaccination.

This study has several limitations that should be considered when interpreting the results. First, the sample size of patients with AIH was limited because the prevalence of AIH was not high, and in the chaos that followed the spread of COVID-19, recruiting a large number of patients to participate in this study was difficult. Second, the results of this study may be biased by measured and unmeasured confounding factors because this study was retrospectively performed in a hospital in Japan and all participants were Japanese. Third, the control group included regular employees at the hospital. Therefore, although a relatively large number of healthy young or middle-aged women were included, the possibility that they were taking immunomodulatory medications or had malignant diseases was not necessarily ruled out with certainty. Finally, information on antibody titers after the third or fourth vaccination has not been collected.

Despite these limitations, we successfully demonstrated that the anti-SARS-CoV-2 spike antibody concentration in the AIH group was significantly lower than that in the control group until 6 months after the second vaccination. In addition, the proportion of patients for whom the vaccine provided little or very limited, if any, symptomatic relief from viral infection was significantly higher in the AIH group. Contrastingly, no cases of serious adverse events associated with vaccination, including severe liver injury, were observed in the study population, which confirms the safety and efficacy of vaccination. 

Since the battle between humans and COVID-19 will probably continue for a long time, continued and widespread vaccination, particularly for patients for whom medical immunomodulation is essential, is desirable.

## Figures and Tables

**Table 1:** Clinical profiles of participants in this study.

	Originally Registered Cases (n = 831)			Propensity Score Matched Cases (n = 30)		
	Controls (n = 809)	AIH Patients (n = 22)	*p*	Controls (n = 15)	AIH Patients (n = 15)	*p*
Age (years)	39.0 [28.0–49.0]	65.0 [61.3–79.0]	<0.01	60.0 [54.0–68.0]	61.0 [54.0–65.0]	0.86
Sex (male/female)	186/623	4/18	0.79	2/32	3/12	1.00
Prednisolone (yes/no)	no data	16/6	-	no data	11/4	-
Prednisolone (mg/day)	no data	5.5 [5.0–6.0]	-	no data	6.0 [5.0–6.5]	-
Azathioprine (yes/no)	no data	4/18	-	no data	3/12	-
Azathioprine (mg/day)	no data	75.0 [75.0–93.8]	-	no data	75.0 [75.0–112.5]	-
anti-allergic agents (yes/no)	no data	0/22	-	no data	0/15	-

AIH, autoimmune hepatitis.

## Data Availability

All the data used to support the findings of this study are included within the article.

## Supplementary Materials

The following supporting information can be downloaded at: https://www.mdpi.com/article/10.3390/jcm12165411/s1. Figure S1: Relations between sex and humoral vaccination response in the study population. (a) Humoral vaccination response in healthy controls 12 weeks after the second vaccination. (b) Humoral vaccination response in healthy controls 26 weeks after the second vaccination. BAU, binding anti-body units.

## Abbreviation

AIH, autoimmune hepatitis; BAU/mL, binding antibody units per milliliter; COVID-19, coronavirus disease 2019; HC, healthy control; NS, not significant; SARS-CoV-2, severe acute respiratory syndrome coronavirus type 2.
