# Supplementary figures and images for: SARS-CoV-2 Vaccination Response in Japanese Patients with Autoimmune Hepatitis: Results of Propensity Score-Matched Case–Control Study
Source: J Clin Med. 2023 Aug 20;12(16):5411. doi: 10.3390/jcm12165411 (PMC10455609; doi:10.3390/jcm12165411)

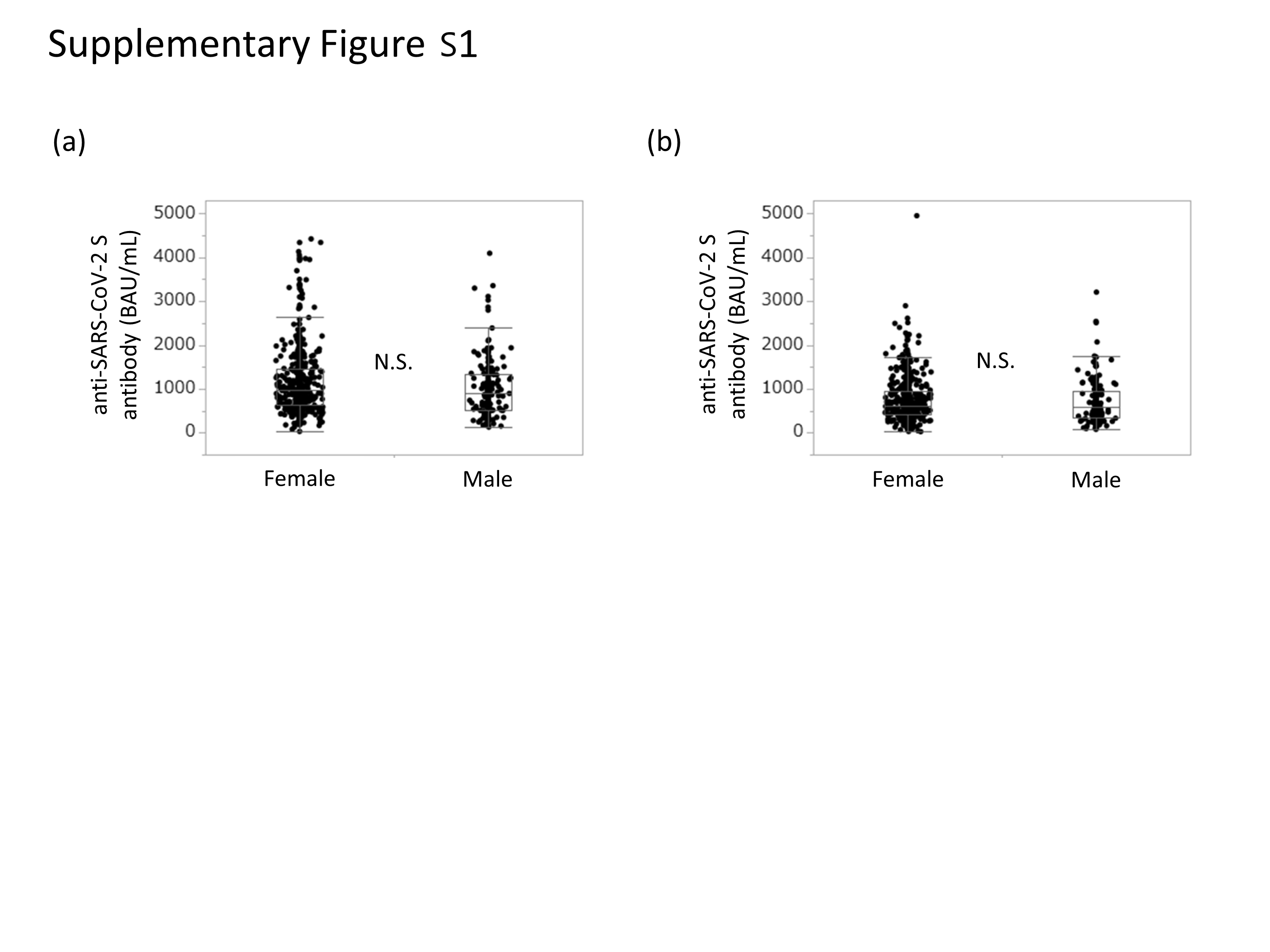

Supplement: Supplementary file 1 [file jcm-12-05411-s001.zip › Sup. Fig.1.tif]
